# Rural–Urban Disparities in COVID-19 Vaccine Uptake and Associated Mortality and Cardiovascular Disease Outcomes in the United States

**DOI:** 10.3390/vaccines13080861

**Published:** 2025-08-14

**Authors:** Bailey Smith, Fahad Farakh, Asma Hanif, Javed H Tunio, Shumaila Nida Javed Tunio

**Affiliations:** 1 Department of Biomedical Sciences, Kentucky College of Osteopathic Medicine, University of Pikeville, Pikeville, KY 41501, USA; baileysmith@upike.edu; 2 Faculty of Medicine, Georgian National University, 0144 Tbilisi, Georgia; ffrakh@seu.edu.ge; 3 Department of Restorative Sciences, College of Dentistry, Kuwait University, Jabriya 13110, Kuwait; asmaa.haneef@ku.edu.kw; 4 Department of Internal Medicine, Carver College of Medicine, University of Iowa, Iowa City, IA 52242, USA; javed-tunio@uiowa.edu

**Keywords:** COVID-19 vaccine, cardiovascular health, COVID-19 mortality inequality, rural community, vaccine hesitancy

## Abstract

**Background:** The COVID-19 pandemic magnified long-standing health disparities in the United States, particularly among rural, disadvantaged populations. These communities experience greater barriers to healthcare access, a higher prevalence of chronic illness, and increased vaccine hesitancy factors that collectively contribute to poorer health outcomes. **Methods:** This narrative review examines rural–urban disparities in COVID-19 vaccine uptake and their impact on mortality, with a focus on cardiovascular disease (CVD) outcomes. We synthesized the peer-reviewed literature, CDC data, and U.S. Census reports to assess factors contributing to vaccine hesitancy, vaccination coverage, COVID-19-related mortality, and CVD mortality trends. **Results:** Rural residents were less likely to initiate COVID-19 vaccination, showed greater vaccine hesitancy, and experienced higher rates of both COVID-19 and CVD mortality. These disparities were further driven by safety concerns surrounding mRNA technology, misinformation, infrastructural barriers, and sociodemographic factors including political affiliation, education, poverty, and religion. Notably, pre-existing CVD increased vulnerability to severe COVID-19 outcomes in rural communities. **Conclusions:** Expanding vaccination efforts and improving healthcare infrastructure are essential for addressing these widening health inequities. Future public health strategies should prioritize culturally tailored interventions and rural-specific outreach to reduce vaccine hesitancy and improve mortality outcomes in underserved populations.

## 1. Introduction

Coronavirus disease 2019 (COVID-19) appeared in the United States in early 2020 and quickly became one of the leading causes of death nationwide [1]. By April 2023, over 104 million cases and 1.1 million deaths were reported in the U.S. alone [2]. During the same period, approximately 81.3% of the population had received at least one dose of a COVID-19 vaccine [1,3], significantly reducing hospitalizations and deaths. It is estimated that within the first two years of availability, COVID-19 vaccination prevented over 3.2 million deaths in the United States [4].

Despite the demonstrated effectiveness of the vaccines, significant disparities in vaccination rates have persisted, particularly between urban and rural populations [5]. Rural counties have consistently reported lower vaccine uptake, with higher levels of vaccine hesitancy and limited access to healthcare infrastructure contributing to this gap [5,6]. Compounding these challenges, rural populations often experience a greater burden of chronic illnesses, especially cardiovascular disease (CVD), a major risk factor for severe COVID-19 outcomes [2,7,8]. As a result, rural communities have faced disproportionately high rates of COVID-19-related mortality [5,6].

In parallel, rural areas also saw an increase in CVD-related deaths during the pandemic [9,10]. In the years leading up to the COVID-19 pandemic, the gap in CVD mortality between rural and urban areas had been narrowing; however, this trend reversed with the emergence of COVID-19, and CVD mortality disparities sharply increased [9,11].

Given that rural populations are at a significantly higher risk for severe COVID-19 complications due to elevated rates of CVD and lower vaccine uptake, it is critical to understand the underlying causes of vaccine hesitancy in these communities [12,13]. This narrative review addresses a central question: How have safety concerns, infrastructural barriers, and sociodemographic characteristics influenced vaccine hesitancy in rural America?

To answer this, we review the existing literature and epidemiological data to examine the drivers of vaccine hesitancy in rural populations. We argue that improving vaccination rates in these areas requires targeted outreach that addresses the unique challenges and beliefs of rural residents [14,15]. In doing so, this review contributes to the broader effort to reduce COVID-19 and CVD mortality disparities and strengthen pandemic response strategies in underserved areas.

## 2. Categorization of Rural and Urban Populations in the U.S.

### Defining Rurality vs. Urbanity

Defining “rurality” in the United States is inherently complex, as there is no single, standard definition used across all federal agencies [16]. For context, the U.S. Census Bureau employs a classification system in which “urban areas” have 50,000 or more residents and “urban clusters” include between 2500 and 49,999 residents, while all remaining areas are “rural” [16]. These population estimates are released in the U.S. Decennial Census every 10 years [17]. In contrast, the Office of Management and Budget (OMB) classifies counties as “metropolitan” for urban areas with 50,000 or more residents, “micropolitan” counties as having between 10,000 and 49,999 residents, and “non-core” describes areas without a cluster of at least 10,000 people [16]. The term “nonmetro” often describes a rural county that does not have an urbanized area with 50,000 or more people [16]. Other agencies, such as the Health Resources and Services Administration (HRSA), apply more nuanced geographic tools like the Rural–Urban Commuting Area (RUCA) codes, which incorporate measures of population density, urbanization, and commuting patterns to identify varying degrees of rurality within and across counties [18]. For clarity, this narrative review provides information on which method of rurality was used when providing the background of rural–urban sociodemographic comparisons and acknowledges the limitations of the variable definition of rural.

## 3. A Background of Rural–Urban Sociodemographic Composition

### 3.1. Rural vs. Urban County Race Profiles

**Figure 1:**
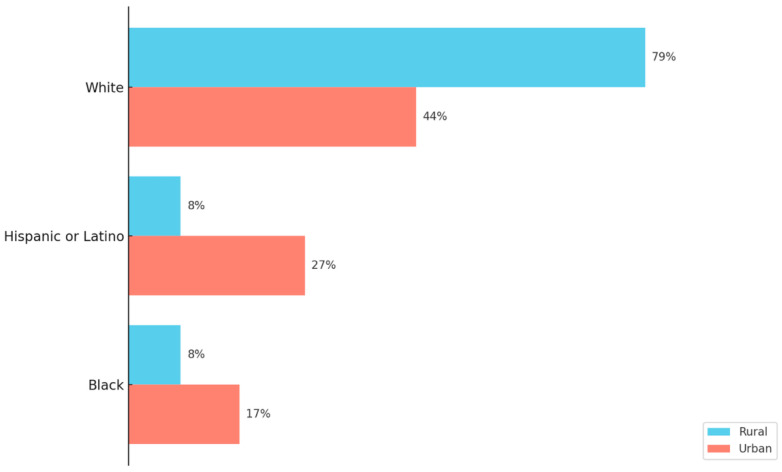
A Comparison of Rural and Urban County Race Profiles. This figure illustrates the race–ethnic profiles with the largest composition differences between rural and urban counties. The data originates from the Pew Research Center analysis of the 2012–2016 American Community Survey data and uses the National Center for Health Statistics Urban–Rural Classification system for counties [22]. Rural areas are shown to be predominately White and less diverse than urban counties [22].

To comprehensively understand rural vaccine uptake disparities, it is important to consider the demographic makeup of rural populations [19,20]. According to the 2020 U.S. Decennial Census, approximately 46 million individuals—about 14% of the total U.S. population—reside in rural areas [21,22]. In comparison, 98 million Americans were found to live in urban counties [22]. While the census data confirmed that rural America remains predominately White, it also highlighted a notable increase in racial and ethnic diversity since the 2010 Census [21]. However, urban counties are becoming more diverse at a much faster pace than rural counties [21,22]. Overall, as seen in Figure 1, when comparing rural and urban county population composition, the largest differences are among White, Hispanic, and Black residents [21,22].

### 3.2. Rural vs. Urban County Age Profiles

**Figure 2:**
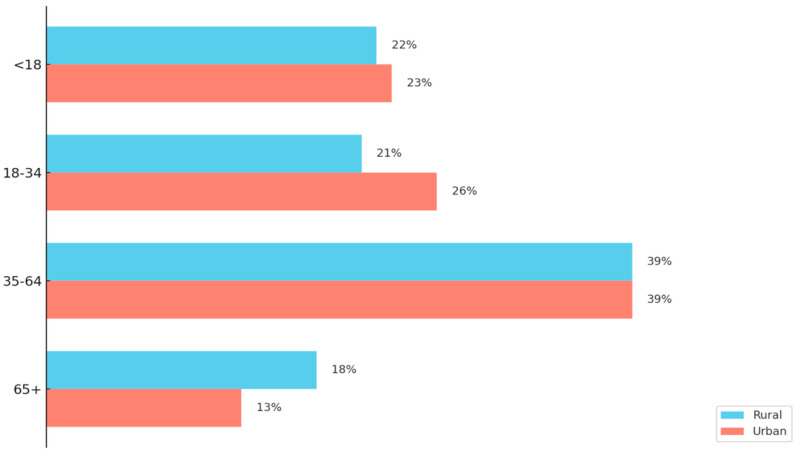
A Comparison of Rural and Urban County Age Profiles. This figure illustrates the age profile differences between rural and urban counties. The data originates from the Pew Research Center analysis of the 2012–2016 American Community Survey data and uses the National Center for Health Statistics Urban–Rural Classification system for counties [22]. Rural populations are shown to have a larger composition of those aged 65 and older than urban counties. In addition, the 18–34 age range is shown to be less present in rural areas compared to urban areas [22].

As the demographic landscape of rural America continues to evolve, recent data seen in Figure 2 highlight a growing imbalance in age distribution that poses significant implications for workforce sustainability and healthcare infrastructure [22,23]. In 2024, the U.S. Department of Agriculture released a report, using the “nonmetro” definition of rural, and found rural counties are experiencing a shift in age profile as there is a growing concentration of both younger (under 15) and older (65 and over) populations, while the working-age population continues to shrink [23]. Between 2010 and 2023, the number of rural residents aged 15–64 declined by over 2 million, whereas the population aged 65 and older rose from 7.4 million to 9.7 million [23]. This aging trend has led to a near tripling of rural “older age counties”—those where 20% or more of the population is over 65 [23]. In 2023, rural counties had significantly higher dependency ratios than urban counties, with some areas averaging 72 dependents (youth and elderly) per 100 working-age individuals [23].

### 3.3. Rural vs. Urban County Poverty, Education, and Employment

**Figure 3:**
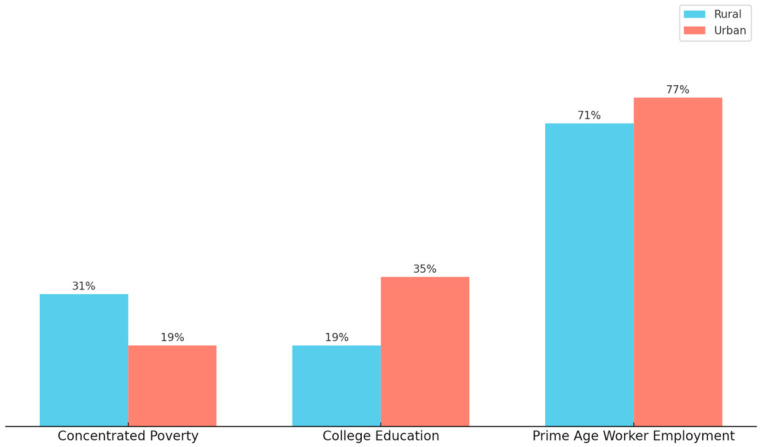
A Comparison of Rural and Urban County Concentrated Poverty, College Education, and Prime Age Worker Employment. This figure illustrates the concentrated poverty, education, and employment differences between rural and urban counties. The data originates from the Pew Research Center analysis of the 2012–2016 American Community Survey data and uses the National Center for Health Statistics Urban–Rural Classification system for counties [22]. Concentrated poverty is a measurement of where at least a fifth of the population in a county is poor and prime age workers are defined by the source to be those 25 to 54 years old [22]. Rural areas are shown to be composed of higher concentrated poverty, less individuals obtaining a college education, and where those of working age are less likely to be employed when compared to urban counties [22].

Rural communities in the United States face distinct socio-economic challenges compared to their urban counterparts, particularly in the areas of poverty, education, and employment [22,24]. While poverty rates are slightly higher in rural areas compared to urban areas, the concentrated poverty difference is much bigger, with 31% of rural counties having at least one-fifth of their population living in poverty, compared to 19% of urban counties [22]. Also, educational attainment lags in rural areas, where only 19% of residents have a bachelor’s degree or higher, compared to 35% in urban counties [22]. In addition, employment disparities are pronounced as 71% of rural residents, aged between 25 and 54, are employed, compared to 77% in urban areas [22]. These combined trends, seen in Figure 3, underscore the widening economic and educational gaps between rural and urban regions.

## 4. A Brief History of COVID-19 Vaccine Development

In January 2020, SARS-CoV-2 was isolated and identified as the causative agent of a pneumonia outbreak in Wuhan, China [25]. The viral genome was sequenced and publicly shared, providing a critical foundation for vaccine development [26]. In February 2020, the three-dimensional structure of the SARS-CoV-2 spike (S) protein was determined, identifying it as the key mediator of viral entry via the angiotensin-converting enzyme 2 (ACE2) receptor and the primary target for neutralizing antibody responses [25].

Building on prior research on SARS-CoV and MERS-CoV, vaccine development efforts rapidly accelerated between March and April of 2020 [26]. Multiple platforms were explored, including mRNA vaccines encoding the spike protein, viral vector vaccines delivering the spike gene, protein subunit vaccines containing purified spike fragments, and inactivated virus vaccines using chemically treated SARS-CoV-2 particles [26]. Then, preclinical testing in animal models evaluated vaccine safety, immunogenicity, and the induction of neutralizing antibodies and T-cell responses [26,27].

Early-phase clinical trials began in July 2020, proving that vaccine candidates such as Moderna’s mRNA-1273 and Pfizer-BioNTech’s BNT162b2 elicited strong immune responses with acceptable safety profiles [27,28]. By September, large-scale Phase III trials were underway globally, revealing approximately 95% efficacy for the mRNA vaccines (Pfizer-BioNTech, New York, NY, USA and Moderna, Cambridge, MA, USA) and 66% efficacy for the adenoviral vector-based Ad26.COV2.S vaccine (Johnson & Johnson-Janssen, New Brunswick, NJ, USA), and preventing moderate to severe COVID-19 [28,29,30]. Following rigorous review, regulatory agencies granted Emergency Use Authorizations (EUAs) in December 2020 for the Pfizer-BioNTech and Moderna vaccines, initiating mass vaccination campaigns and representing a significant milestone in pandemic control [1,28].

**Figure 4:**
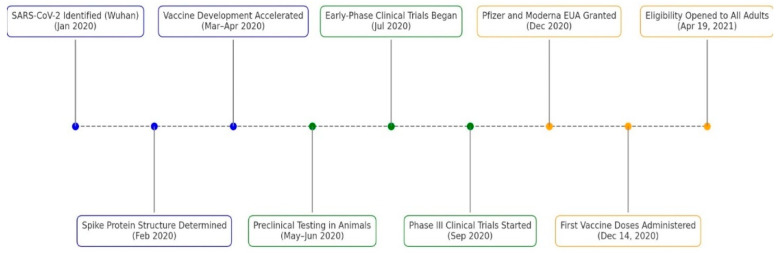
Timeline of COVID-19 Vaccine Development Milestones (2020–2021). Blue markers show early development events, green markers show clinical trial phase milestones, and orange markers denote public vaccine authorizations and rollout. Each event is labeled with its description and date [1,25,26,28].

After the EUA for Pfizer-BioNTech’s and Moderna’s vaccine, states across the United States began administering doses within days, guided by Centers for Disease Control and Prevention (CDC) recommendations and state-specific distribution plans [1]. By December 21, all states, Washington D.C., and U.S. territories had begun vaccine distribution [1]. Throughout January 2021, eligibility gradually broadened to include frontline essential workers, individuals over 65, and those with high-risk medical conditions [1]. However, access varied across states due to differences in infrastructure and vaccine supply [1,31]. By 19 April 2021, all states were directed by the federal government to open vaccine eligibility to all adults aged 16 and older, marking a pivotal point in the nationwide immunization effort [1]. Overall, the timeline (Figure 4) from virus molecular structural identification to EUA was approximately 1 year [1,26].

## 5. Rural–Urban Health Disparities in the U.S.

### A History of Poor Cardiovascular Disease Health Outcomes in Rural Communities

Over the past several decades, rural populations have consistently experienced worse health outcomes than their urban counterparts, including higher rates of chronic disease and premature mortality [11,12,19]. The COVID-19 pandemic exacerbated disparities in socioeconomic conditions and healthcare resources in rural counties, contributing to poor health outcomes and reinforcing previous patterns of mortality disparities [11,19].

**Figure 5:**
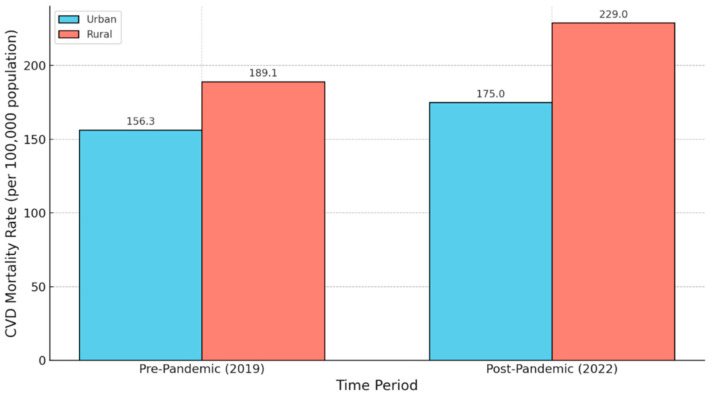
Cardiovascular Disease Mortality in Rural and Urban Areas (Pre- and Post-Pandemic). This figure illustrates the age-adjusted mortality rates from cardiovascular disease per 100,000 population in rural and urban areas of the United States before (2019) and after (2022) the onset of the COVID-19 pandemic. Pre-pandemic, rural areas exhibited a higher CVD mortality rate (189.1 deaths per 100,000) compared to urban areas (156.3 deaths per 100,000) [45]. Following the pandemic, mortality rates increased in both populations but rose more sharply in rural areas (229.0 deaths per 100,000) relative to urban areas (175.0 deaths per 100,000), widening the disparity [9]. These findings highlight the exacerbation of rural–urban health disparities in cardiovascular outcomes during the COVID-19 pandemic.

Cardiovascular disease (CVD) has long been a leading contributor to excess mortality in rural areas [11,12,32]. From 1999 to 2019, rural counties experienced higher age-adjusted CVD mortality rates compared to urban counties—a disparity that had been slowly narrowing until 2019 [11,12]. Between 2010 and 2022, cardiovascular death rates among adults aged 25–64 rose by approximately 21% in rural areas, compared to a 3% rise in large metropolitan areas [9]. These trends sharply accelerated following the emergence of COVID-19 [2,11,12]. From 2020 to 2022, CVD mortality increased by 8.3% in rural regions compared to 3.6% in urban areas [9,10]. By 2022, the national age-adjusted mortality rate (AAMR) for CVD reached 434.6 deaths per 100,000 population—returning to levels not seen since a decade earlier [10]. This surge, seen in Figure 5, coincided with strained rural healthcare systems, delayed care, and increased barriers to chronic disease management [19].

The longstanding cardiovascular health disparities between rural and urban populations contributed to the disproportionate COVID-19 mortality experienced by rural communities [8,33,34]. Since individuals with underlying heart disease face a markedly higher risk of severe outcomes following COVID-19 infection, rural areas with elevated CVD were particularly burdened during the pandemic [2,8,35]. One study found that mortality was 52% higher in rural counties than in urban counties [34]. Another analyzed over 1 million COVID-19 patients across 44 hospital systems from January 2020 to June 2021 and found that rural patients had a 36% higher mortality rate than their urban counterparts [36]. In addition, a cohort study in North Carolina reported that 16.5% of patients from rural areas died or were discharged to hospice, compared to 13.3% in urban populations [37]. The relationship between poor COVID-19 health outcomes and a population with elevated CVD is evident in a study that found an in-hospital mortality of 7.62% among patients without CVD, compared to a striking 69.44% among those with pre-existing cardiac disease [38]. These findings underscore the role that pre-existing cardiovascular conditions had in COVID-19 mortality gaps between rural and urban counties [34,35,36,37,38].

Despite the increased vulnerability of rural communities, vaccination against COVID-19 played a vital role in reducing hospitalizations and deaths across all populations, including individuals with pre-existing cardiac disease [39,40]. For patients with CVD, vaccination serves as a critical safeguard, offering essential protection for those most at risk to severe complications and death [39,40]. Observational analyses of county-level data from 2558 counties—representing about 300 million individuals and 80% of the national population—found that each 10% increase in vaccination coverage corresponded to an 8% reduction in COVID-19 mortality between December 2020 and December 2021 [41]. Additional modeling by the National Institutes of Health estimated that vaccination efforts prevented 140,000 deaths by May 2021, while a separate study from Yale University projected approximately 279,000 deaths and 1.25 million hospitalizations were averted by July 2021 [42,43]. Notably, most deaths during this period occurred among unvaccinated and elderly individuals [44]. Since many studies found disparities between rural and urban population vaccination uptake, expanding future vaccination among high-risk rural populations is imperative in addressing the COVID-19 mortality disparities [5,6,8,41].

## 6. Exploring Contributing Factors to Vaccine Hesitancy

### 6.1. Vaccine Hesitancy in Rural Communities

To fully grasp the roots of the COVID-19 vaccination disparities, examining the role of vaccine hesitancy is critical—one of the key behavioral and structural factors contributing to lower vaccination rates in rural communities [15,33]. The World Health Organization defines vaccine hesitancy as the “delay in acceptance or refusal of vaccination despite availability of vaccination services [46].” However, the concept remains inconsistently applied across the literature [47]. In a systemic review, vaccine hesitancy was found to not be a stable personal trait but a dynamic, context-specific phenomenon that varies by time, place, and vaccine [47]. Many factors can contribute to vaccine hesitancy, such as safety concerns, infrastructural barriers, and sociodemographic characteristics [13,19,47,48]. A retrospective analysis of vaccination data from 29 U.S. states showed that in April 2021, following expanded vaccine eligibility, 53.3% of rural adults and 42.2% of urban adults remained unvaccinated [6]. By December 2022, 32.5% of rural adults remained unvaccinated compared to 19.3% of their urban counterparts [6]. Similarly, CDC surveillance data from December 2020 to January 2022 found that 58.5% of adults in rural areas had received at least one vaccine dose, compared to 75.4% in urban areas [5]. These vaccine disparities extend to pediatric populations; as of December 2022, vaccination coverage among children was 3.4% in rural counties versus 10.5% in urban counties [49]. Addressing rural–urban COVID-19 vaccine disparities requires targeted interventions that address the factors contributing to vaccine hesitancy [15]. Without such focused efforts, vaccine hesitancy will continue to contribute to the exacerbation of COVID-19 mortality inequities across the United States [15].

### 6.2. Safety Concerns About mRNA Vaccine Technology

Surveys indicate that individuals perceiving COVID-19 vaccines as unsafe are more likely to reside in rural areas [15,33]. Since misinformation about the safety of COVID-19 vaccination can fuel vaccine hesitancy, it is important to educate and address apprehension to restore vaccine confidence and improve future vaccine uptake [33]. Among some individuals, the Pfizer-BioNTech and Moderna vaccines have prompted safety concerns due to their prompt release and their distinction as the first mRNA-based vaccines approved for public use [27,33]. However, while these were the first mRNA vaccines widely deployed, the underlying messenger RNA (mRNA) technology has been under development and rigorous study for decades [26,27]. Research into mRNA platforms began in the early 1990s and accelerated in the 2000s, with promising applications in cancer immunotherapy and vaccine candidates for infectious diseases such as Zika, rabies, and influenza [26,50]. Over the years, key challenges such as stabilizing synthetic mRNA, refining its translation, and encapsulating it in lipid nanoparticles have been addressed for efficient delivery into human cells [50]. These foundational advances enabled the rapid, yet scientifically sound, development of COVID-19 vaccines [26,50]. Far from being experimental or untested, the mRNA approach used for COVID-19 vaccines represents the culmination of decades of biomedical innovation and peer-reviewed research, with well-established safety and efficacy profiles prior to emergency use authorization [26,50].

### 6.3. Safety Concerns About COVID-19 Vaccine Adverse Reactions

Another source contributing to increased COVID-19 vaccine hesitancy is the widespread circulation of misinformation about rare adverse reactions reported after vaccination [33,51]. This misinformation, particularly when amplified through social media platforms, has contributed to a broader transformation of vaccine hesitancy [48]. Although mRNA vaccines showed strong safety profiles in large clinical trials, post-marketing surveillance found rare adverse events that raised public concern—most notably, cases of myocarditis in male adolescents [28,29,51]. In October 2021, the U.S. Centers for Disease Control and Prevention (CDC) formally acknowledged myocarditis as a rare adverse event associated with mRNA vaccines, particularly after the second dose in young males [52,53]. Subsequent analyses further clarified that the risk of vaccine-associated myocarditis is most pronounced following the second dose of mRNA COVID-19 vaccines, particularly among young adult males [51,52]. One cohort study reported an incidence of 0.8 cases per 1 million first doses compared to 5.8 cases per 1 million second doses within a 10-day post-vaccination window, with an incidence rate ratio (IRR) of 0.38 after the first dose and 2.7 after the second [54]. Similarly, an analysis of CDC surveillance data from December 2020 to June 2021 found that 76% of reported myocarditis cases occurred after the administration of the second dose [52]. More research corroborated these findings, reporting myocarditis incidence rates of 3.37 and 21.22 per 100,000 individuals following the first and second doses, respectively [55]. While these studies confirm an increased risk of myocarditis following the second mRNA vaccine dose—particularly in young males—it is critical to evaluate this risk by comparing it to the higher incidence of cardiac injury associated with virus infection [52,54,55].

### 6.4. Addresing Safety Concerns: Improved Education on the Risks from Vaccination vs Infection

It is important to educate the public on the contextualized risks of COVID-19 vaccination to address safety concerns and reduce the greater vaccine hesitancy in rural areas. Research has evaluated the relative risk of developing myocarditis following COVID-19 vaccination compared to SARS-CoV-2 infection and found that infection without the vaccine is more likely to cause harm than from the vaccine itself [35,56]. One study reported a risk ratio of 3.24 for myocarditis associated with vaccination, while the risk following COVID-19 infection was higher at 18.28 [56]. A comprehensive review further highlighted that the incidence of COVID-19-associated cardiac injury or myocarditis is estimated to be 100 times greater than the incidence of myocarditis following mRNA vaccination [35]. Notably, outcomes among vaccine-associated myocarditis cases have been overwhelmingly favorable; over 90% of affected individuals achieved full recovery [51]. One study documented seven cases of clinical myocarditis following the Pfizer-BioNTech vaccine, all of whom recovered rapidly [57]. Moreover, another reported eight deaths attributable to vaccine-associated myocarditis globally, reflecting a survival rate exceeding 99% [35]. Given the significantly higher health risks associated with SARS-CoV-2 infection—even among younger populations—the risk-benefit profile strongly favors vaccination [51,56,57].

### 6.5. The Impact of Infrastructural Barriers and Inaccessible Health Care in Rural Communities

Limited accessibility to the COVID-19 vaccine also likely contributes to vaccine hesitancy and uptake disparities, as rural communities in the United States face significantly more barriers to healthcare access [37]. Rural areas often have fewer healthcare facilities, a limited number of medical professionals, and longer travel distances to care [8,37]. As a result, individuals in rural counties must travel farther to receive vaccinations or medical treatment than their urban counterparts [5,37]. The accessibility burden reduces the convenience of vaccination and can prevent or discourage individuals from seeking medical care [37]. Overall, this geographic isolation imposes an added structural barrier to rural vaccine uptake and prompt disease management, therefore contributing to the COVID-19 and CVD mortality disparities [37].

### 6.6. Addressing Infrastructural Barriers: Improved Vaccine Access

Evidence shows that vaccine uptake increased following the expansion of local vaccine sites; notably, one evaluation reported a 73% increase in vaccinations after additional sites were introduced [58]. These findings underscore how infrastructural inequities—unique to rural settings—may have contributed to elevated case fatality rates during COVID-19 surges [37]. Strengthening rural healthcare infrastructure is essential for future public health preparedness, and reducing travel distances through mobile clinics or localized vaccine hubs may offer a viable strategy to improve access and mitigate health disparities [58].

### 6.7. A Brief Review of How Sociodemographic and Cultural Factors Influence Vaccine Hesitancy

In addition to safety concerns and infrastructural barriers, numerous sociodemographic and cultural factors—such as race, gender, age, education, socioeconomic status, political affiliation, and religion—have been shown to influence COVID-19 vaccine hesitancy [8,47,48]. As of early 2022, rural Americans remained the least likely demographic to initiate COVID-19 vaccination [44]. A 2023 systematic review reported that concerns regarding vaccine safety and potential side effects were particularly elevated among racial and ethnic minority groups [47]. While hesitancy varies across racial and ethnic lines, multiple studies have found that Black Americans consistently exhibit the highest levels of COVID-19 vaccine hesitancy [8,44,47]. Contributing factors include longstanding distrust in the medical establishment and underrepresentation of minority populations in vaccine clinical trials [44,47,48]. Gender and age have also been associated with differing attitudes toward vaccination, with women more likely to express hesitancy than men, and younger adults more hesitant than older individuals [47,59]. Educational attainment and higher socioeconomic status have generally been linked to more favorable attitudes toward vaccination and, during the pandemic, rural counties with lower socioeconomic status exhibited both lower vaccine uptake and disproportionately higher COVID-19 mortality [7,8,47,59]. Vaccine hesitancy in the United States has increasingly followed political fault lines [47,48]. By mid-2021, political partisanship had become one of the strongest predictors of vaccine hesitancy, surpassing race, age, and income [48]. Republican-leaning counties demonstrated persistently lower uptake despite increased availability [48]. Additionally, religious beliefs have been found to influence vaccination behavior [47]. A multi-country analysis of 195 regions concluded that areas with higher levels of religiosity tended to have lower COVID-19 vaccination rates [60]. Together, these intersecting factors underscore the complexity of vaccine hesitancy and highlight the need for multifaceted, community-specific strategies to improve vaccine confidence and coverage.

### 6.8. Sociodemographic and Cultural Factors’ Influence on Increased Rural Vaccine Hesitancy

Rural communities in the United States exhibit higher levels of COVID-19 vaccine hesitancy, influenced by a constellation of interrelated factors [6,14,15]. First, political affiliation—especially a higher share of Republican voters—emerged as the strongest county level predictor of low vaccination rates, even when controlling for education, income, and demographics [48,61]. Rural counties are disproportionately Republican leaning, aligning them with elevated hesitancy patterns observed nationally [22,61]. Second, lower levels of educational attainment and socioeconomic status, which are more prevalent in rural settings, correlate with reduced vaccine acceptance and uptake [14,15,20,22]. Third, higher poverty rates and limited healthcare infrastructure in rural regions restrict both access to vaccines and health literacy, intensifying distrust and resistance [14,15,20,22]. Fourth, religious and cultural identities, including evangelical or conservative Christian affiliations common in many rural areas, are independently associated with lower vaccine uptake [22,60]. Taken together, these socio-political, economic, and cultural factors help explain the persistence of higher vaccine hesitance in rural America.

## 7. Limitations

This narrative review has several limitations. First, the data sources used vary in collection methods and reporting periods, which may affect comparability across datasets. Second, rural–urban classifications differ slightly between sources, introducing potential misclassification bias. Third, as a descriptive review, this study does not establish causality between vaccine hesitancy, vaccine uptake, and mortality outcomes. Fourth, this review does not evaluate specific public health strategies or interventions that may have been implemented to address vaccine disparities, which could further contextualize the observed patterns. Finally, generalizability may be limited due to state-level policy variations, differences in healthcare infrastructure, and reporting inconsistencies across regions. Despite these limitations, this review provides important insights into the persistent rural–urban disparities in vaccine uptake, hesitancy, and health outcomes, highlighting critical areas for future research and public health intervention.

## 8. Conclusion

The COVID-19 pandemic exacerbated longstanding health disparities in the United States, disproportionately affecting rural communities. Despite the proven efficacy of COVID-19 vaccines in reducing mortality, persistent vaccine hesitancy—fueled by safety concerns, infrastructural barriers, and sociodemographic characteristics—continue to hinder vaccination efforts among vulnerable rural populations.

To fully address safety concerns, further research is needed to clarify the mechanisms underlying rare adverse events, such as myocarditis, particularly in young males following the second dose of mRNA-based vaccines. However, it is essential to educate the public on these findings: myocarditis following vaccination is rare and typically self-limiting, while the risk of severe cardiac complications or death from SARS-CoV-2 infection is substantially higher.

Our review underscores that rural residents were significantly less likely to initiate vaccination and experienced disproportionately higher COVID-19 mortality rates, especially among those with pre-existing cardiovascular disease. In addition, we highlight that during the pandemic, the previously waning CVD mortality disparities increased in rural counties. Vaccination remains one of the most effective tools for preventing severe outcomes, and increasing uptake in rural areas is vital to closing persistent mortality gaps. The benefits of COVID-19 vaccination far outweigh the associated risks, particularly for high-risk and underserved populations.

Closing the rural–urban vaccination gap is critical not only for managing the ongoing impact of COVID-19 but also for strengthening preparedness for future public health threats. Targeted interventions are urgently needed to enhance vaccine uptake, expand healthcare access, and deliver culturally tailored health education. Addressing these barriers will protect vulnerable rural populations, mitigate health inequities, and build a more resilient healthcare system. Inaction risks perpetuating avoidable mortality and deepening rural–urban health disparities in future crises.

## Data Availability

No new data was created.
